# Transvenous Lead Extraction Complicated by Lead Breakage: A Predictive Model Based on Analysis of the EXTRACT Registry

**DOI:** 10.3390/jcm15031216

**Published:** 2026-02-04

**Authors:** Michal Joniec, Joanna Stachanczyk, Rafal Gardas, Sylwia Gladysz-Wanha, Eugeniusz Pilat, Anna Drzewiecka, Jolanta Biernat, Andrzej Weglarzy, Wojciech Wanha, Danuta Loboda, Krzysztof S. Golba

**Affiliations:** 1 Department of Electrocardiology, Upper-Silesian Medical Centre in Katowice, Ziolowa 45/47 Str., 40-635 Katowice, Polandkgolba@sum.edu.pl (K.S.G.); 2 Doctoral School of the Medical University of Silesia in Katowice, Poniatowskiego 15 Str., 40-055 Katowice, Poland; 3 Department of Cardiac Rehabilitation, Murcki Hospital, 40-749 Katowice, Poland; 4 Department of Electrocardiology and Heart Failure, Medical University of Silesia in Katowice, Ziolowa 45/47 Str., 40-635 Katowice, Poland; 5 Department of Cardiac Anaesthesia and Intensive Care, Upper-Silesian Medical Centre in Katowice, Ziolowa 45/47 Str., 40-635 Katowice, Poland; 6 Third Department of Cardiology, Upper-Silesian Medical Centre in Katowice, Ziolowa 45/47 Str., 40-635 Katowice, Poland; 7 Department of Cardiology and Structural Heart Diseases, Medical University of Silesia in Katowice, Poniatowskiego 15 Str., 40-055 Katowice, Poland

**Keywords:** transvenous lead extraction, incomplete extraction, lead break prediction

## Abstract

**Background**: The lead breakage (LB) during transvenous lead extraction (TLE) increases procedural complexity, increases the risk of complications, and decreases procedural efficiency. This study aimed to identify protective and risk factors for the breakage of cardiac electronic device leads during extraction. **Methods**: Data were sourced from the EXTRACT prospective registry for TLE procedures conducted between January 2016 and June 2025. A total of 702 consecutive TLE procedures involving 1375 leads were enrolled. Multivariate logistic regression was used to identify independent protective and risk factors and develop a model to predict the occurrence of LB during TLE. **Results**: In the analysed group, 56 (7.98%) of 702 TLE procedures were disrupted by the breakage of at least one lead. The model showed a lower lead breakage rate in procedures when an atrial lead was simultaneously extracted, a locking stylet was used, and when the procedure was conducted in older patients or those who had undergone prior cardiac surgery. Higher risk of LB was proven in the following cases: the extraction of leads implanted a long time ago; the extraction of VDD-type leads; the extraction of abandoned leads; extraction during a prolonged procedure. Occurrence of lead breakage may lead to pericardial effusion requiring intervention, acute kidney injury, or leaving remnants of the leads. **Conclusions**: Lead breakage is an underestimated procedural difficulty that can occur during transvenous lead extraction. In this study, several clinical and procedural variables were independently associated with lead breakage. Abandoned leads, VDD leads, and prolonged procedure time were associated with increased risk. In contrast, older age, use of a locking stylet, atrial lead extraction, prior cardiac surgery, and later year of implantation demonstrated independent protective associations.

## 1. Introduction

Transvenous lead extraction (TLE) from implantable cardiac devices is a well-established method, regardless of the device and lead type [1,2,3,4]. According to AHA/HRS/EHRA guidelines, the minimally invasive removal of all leads in their entirety, with no permanent disabling intraprocedural complications, is crucial for procedural and clinical success in lead management procedures [5,6,7]. Therefore, percutaneous removal is preferred over surgical intervention in most cases [5]. This method even allows for same-day discharge in certain cases [8]. Although the effectiveness and safety of TLE are high, particularly in high-volume centers [9] percutaneous CIED lead extraction is not free of difficulties and complications.

Breakage of lead during extraction is not considered a complication in itself; rather, it is thought of as an unfavorable event or difficulty. However, it increases the complexity and difficulty of the procedure and may lead to life-threatening issues. Especially in patients with infectious TLE, it is crucial to remove all lead fragments to achieve a successful clinical outcome. It should be emphasized that avoiding lead breaking not only enhances the chances of procedural and clinical success but may also help reduce the occurrence of complications and improve the overall safety and effectiveness of TLE.

Many years of performing TLE have yielded numerous publications, which provide statistics on the incidence of specific complications; however, the risk factors for their occurrence remain poorly understood. In turn, knowledge of predictive factors of complications makes it easier to prevent them. Lead breakage, which is not directly classified as a complication, has been described in detail in only two publications [10,11].

This study aimed to identify key factors influencing the breakage of cardiac implantable electronic device leads during extraction, with parameters observed throughout hospitalization, both before and during the procedure. These factors may aid in patient selection and guide decision-making during the procedure.

## 2. Materials and Methods

### 2.1. Patients Population

**Figure 1:**
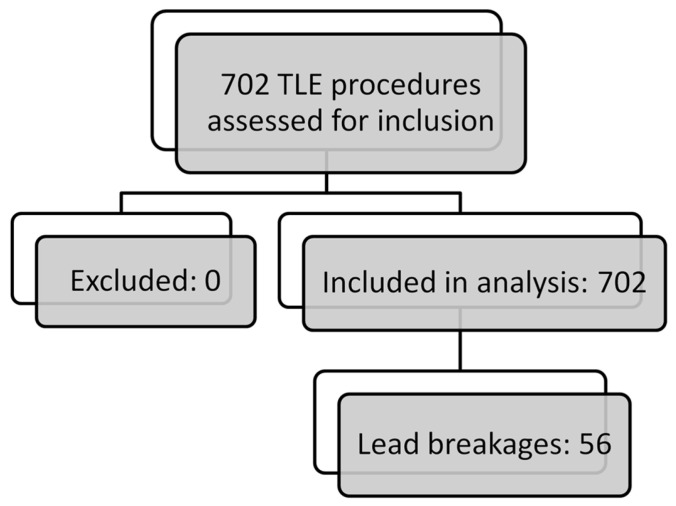
Study flow chart.

Data were collected from 702 consecutive TLEs involving 1375 leads in 684 unique patients. The study was conducted prospectively through the EXTRACT registry for TLE procedures, registered between January 2016 and June 2025 at the Department of Electrocardiology and Heart Failure, Medical University of Silesia, Katowice, Poland (ClinicalTrials.gov ID: NCT05775783, 25 February 2023). According to the definition of TLE proposed by HRS, the registry includes procedures for the extraction of a cardiac electronic device system containing at least one lead whose dwell time is above one year or that requires the use of specialized equipment not usually needed during lead implantation to its removal [6]. There were no exclusion criteria (Figure 1).

### 2.2. Procedure

As described in a previous publication [2], all surgeries at our center were performed in a hybrid operating room by an experienced team consisting of two to three cardiologists, a scrub nurse, a cardiac anesthesiologist, an anesthesiology nurse, an echocardiographer trained in intraoperative TEE, and a radiology technologist. The cardiac surgery team remains on standby during key stages of the procedure. Procedures are performed under general anesthesia with preserved spontaneous breathing. Hospital procedures for procedure preparation and execution are based on current guidelines and best practices. According to the HRS and EHRA expert consensus, “procedural success” is defined as removal of all targeted leads and materials, with the absence of any permanently disabling complications or procedure-related death. In contrast, “clinical success” allows the retention of a small portion (<4 cm) of a lead fragment that does not negatively impact the outcome goals of the procedure when the residual part does not increase the risk of any undesired outcome [6,7]. All procedures were conducted using dedicated Cook Medical equipment, as listed in Table S1. All data were collected from medical records and independently verified by the authors.

### 2.3. Lead Break Definition and Diagnosis

Lead breakage (LB) is defined as a breakage in the continuity of all lead elements, leading to the formation of at least two separate fragments—proximal and distal. If more than two rupture sites occur, a free fragment is formed, which may remain attached to the wall of the cardiovascular system or be released and flow with the bloodstream, most often into the pulmonary circulation. All procedures in which any electrode was broken during extraction were classified as an LB, regardless of the simultaneous occurrence of complications and the final effect of the procedure [11].

LB is diagnosed intraoperatively based on the radiological image and by the extraction of the proximal lead fragment. To date, LB has been classified by the guidelines only indirectly as the retention of a lead fragment that could not be removed during the procedure, regardless of the technique used [7]. Leaving a lead fragment always precludes procedural success; however, in non-infective cases, leaving a fragment shorter than 4 cm does not negatively affect the outcome and can be considered clinical success.

**Figure 2:**
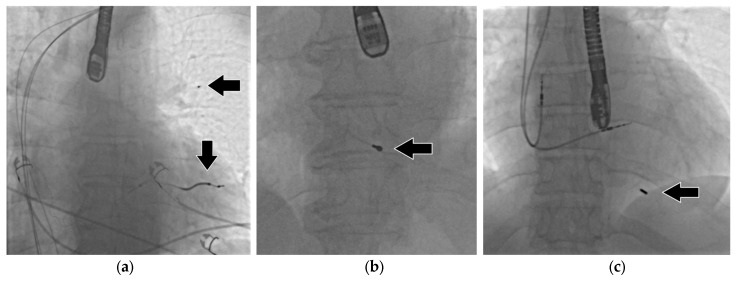
(**a**) Long lead fragment and free-floating lead tip. (**b**) Short lead fragment. (**c**) Tip of the lead.

Kutarski et al. [11] proposed a more precise classification of LB in 2024, as follows: 1. LB with a long (≥4 cm) lead fragment (Figure 2a); 2. LB with a short (<4 cm) lead fragment (Figure 2b); 3. LB with the tip of the lead only (Figure 2c); 4. LB with loss of a free-floating lead fragment or lead tip (Figure 2a). In our opinion, this classification accurately reflects the realities of the TLE procedure and the possibility of removing the torn lead fragment.

### 2.4. Management of Broken Leads

**Figure 3:**
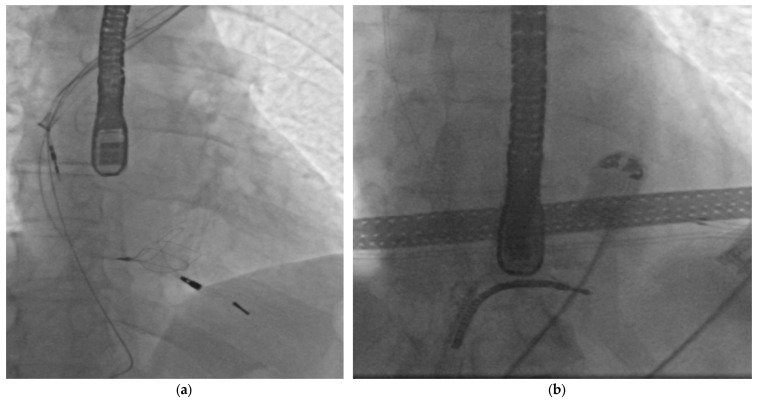
(**a**) Long lead fragment grasped by a basket loop. (**b**) High-voltage lead grasped by a side loop.

The standard procedure in the event of lead stretching or fracture is to attempt to remove the remaining fragments. Depending on the location and number of fractures, different strategies should be used to extract the remaining fragments [12,13]. For leads damaged within the device pocket (before or during the procedure), it is usually sufficient to extend the distal fragment by using a locking stylet or, in the case of an occluded lumen, a lumenless lead extender, and then continue extraction using typical techniques. If a fragment is created that cannot be reached from the pocket, it is necessary to grasp the proximal end using a simple or basket loop with access through the device pocket (Figure 3a). The loop then acts as an extension and stiffener of the lead, allowing the use of mechanical sheaths to complete the extraction. An analogous strategy using jugular vein puncture is acceptable (The “Pisa Technique”), but is not used routinely in our center [14]. If the free end of the lead is inaccessible, a lateral loop via the femoral vein can be used (Figure 3b). Due to the large distance from the puncture site and the large diameter of the instrument, it is not possible to use mechanical sheaths; only simple traction in the caudal direction is available. Sometimes this force vector can be sufficient. Except for patients with infection, removal of a torn lead tip attached to the myocardium was not routinely attempted. It is good practice in patients with old leads or heart failure to use a lateral loop at the very beginning of the procedure. The gripping and countertraction of the lead in the atrium contribute to less tension on the lead in its distal course and less traction on the myocardium.

### 2.5. Statistical Analysis

Continuous data are presented as mean ± SD. The distribution’s normality was confirmed using the Kolmogorov–Smirnov test. The independent two-sample *t*-test was used to compare the two groups. Qualitative data were reported as counts and percentages. Categorical data were analyzed with the chi-square test. A *p*-value < 0.05 was considered statistically significant. Multivariate logistic backward regression was used to identify independent risk factors and develop a model to predict the occurrence of LB during TLE. Both significant variables and those with a *p*-value below 0.1 from the *t*-test or chi-square test were considered candidate variables. The c-statistic, used to assess the model’s discriminating power, and the Hosmer–Lemeshow test, used to evaluate the model’s goodness-of-fit, were employed to assess the model’s predictive power. All reported associations are presented as odds ratios (ORs) with corresponding 95% confidence intervals (95%CI). The odds ratio for continuous variables represents the relative change in endpoint risk per unit increase in the variable. The area under the curve (AUC) for the tested model was determined. MedCalc Statistical Software version 23.1.7 (MedCalc Software Ltd., Ostend, Belgium) and SigmaStat software version 4.0 (Systat Software Inc., San Jose, CA, USA) were used. Additional cross-validation and a calibration plot for the predictive model were performed using Python 3.14.2 with Pandas 2.3.3.

## 3. Results

### 3.1. Incidence of Lead Breakage and Analytical Framework

During the analysed period, 56 (7.98%) out of a total of 702 TLE procedures were disrupted by the breakage of at least one lead.

Due to the large number of analyzed candidate variables as predictors of LB, they were grouped into four blocks: (1) patients’ anamnesis and actual state, (2) patients’ laboratory parameters, (3) characteristics of extracted CIED, and (4) techniques used during the procedure.

### 3.2. Clinical Data of Patients

Univariate analysis of collected anthropometric data, medical history, and basic cardiac parameters revealed a significantly higher rate of lead breaks in younger patients. The mean age of patients who experienced LB was 60.5 ± 15.8 years, compared to 66.7 ± 12.6 years for the group without this event. Of the 245 participants, 21 women were affected by LB (*p* = 0.6707). Similarly, there were no differences in height or weight. Patients who experienced lead breakage during TLE less frequently were diagnosed with coronary artery disease (37.5% in LB group vs. 52.3% with *p* = 0.0334 in the LB-free group), had a history of myocardial infarction (19.6% vs. 35.4%, *p* = 0.0168), had undergone PCI (17.9% vs. 33.9%, *p* = 0.0141) or CABG (1.8% vs. 15.3%, *p* = 0.0055). A lower risk of LB was also confirmed in patients with any of the above circumstances (37.5% vs. 52.8%, *p* = 0.0282). A decrease in the incidence of the analyzed event was observed, accompanied by a decrease in the burden of the above parameters (1.4 ± 1.5 vs. 0.8 ± 1.2, *p* = 0.0046). Previous cardiac surgery, regardless of its extent, was also associated with a lower LB rate (5.4% vs. 23.5%, *p* = 0.0017).

Similarly, patients with peripheral artery disease, atrial fibrillation, and arterial hypertension had a lower incidence of LB (14.3% vs. 27.4%, *p* = 0.0327; 30.4% vs. 44.7%, *p* = 0.0375; 53.6% vs. 68.7%, *p* = 0.0202, respectively), and the use of anticoagulants showed the same trend of association (33.9% vs. 46.7%, *p* = 0.0649). Among the parameters describing the patient’s current cardiac status, both lower NYHA class (mean 0.7 ± 1.1 vs. 1.1 ± 1.1, *p* = 0.0016) and higher left ventricular ejection fraction (51.5 ± 13.7 vs. 44.8 ± 15.5, *p* = 0.0018) are associated with a more frequent occurrence of LB.

An additional analysis of a subgroup of patients who underwent cardiac surgery for any reason revealed a higher percentage of systems with high-voltage leads (47.7% vs. 36.6%, *p* = 0.0118), whereas the pacemaker systems were present in 49.0% of patients who had undergone cardiac surgery in the past, compared to 60.5% without (*p* = 0.0106). Patients who had undergone cardiac surgery in the past also had a higher degree of heart failure, according to the NYHA scale (1.63 ± 1.06 vs. 0.96 ± 1.13, *p* < 0.0001), lower LVEF (38.12 ± 14.20 vs. 47.34 ± 15.26, *p* < 0.0001), and higher coronary artery disease burden (2.48 ± 1.52 vs. 1.00 ± 1.26, *p* < 0.001), Table 1.

### 3.3. Laboratory Parameters

Among laboratory tests routinely performed on admission to the hospital, lower creatinine levels (1.0 ± 0.4 vs. 1.1 ± 0.6 mg/dL, *p* = 0.0253) and d-dimer levels (774.9 ± 1141.9 vs. 1296.2 ± 2555.9 ng/mL, *p* = 0.0306) were associated with a higher incidence of LB. Complete data are listed in Table S2.

### 3.4. Characteristics of the Extracted System

**Figure 4:**
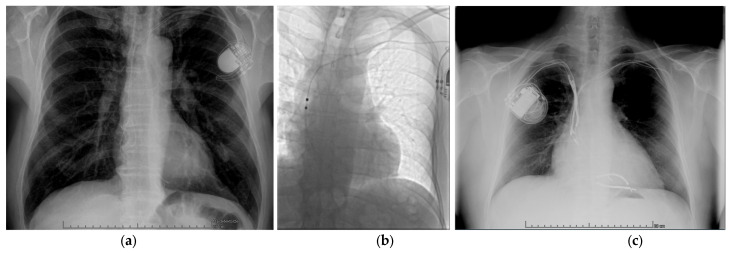
(**a**) Unipolar passive leads. (**b**) VDD lead. (**c**) Many leads after many procedures in one patient.

Both parameters describing the age of the extracted system were connected to the risk of LB during TLE. Systems implanted earlier had a higher risk of developing LB (for 2002.1 ± 8.4 vs. for 2011.9 ± 6.6, *p* < 0.0001). Figure 4a. Similarly, a higher longest dwell time was a cause of LB (18.0 ± 8.3 vs. 8.4 ± 6.5 years, *p* < 0.0001). A higher number of previous interventions in the system was associated with the analyzed event (1.6 ± 1.0 vs. 0.7 ± 0.9, *p* < 0.0001). LB was more likely during the extraction of leads from pacemakers (71.4% vs. 57.9%, *p* = 0.0254) than from high-voltage devices (10.7% vs. 24.8%, *p* = 0.0177).

Univariate analysis revealed that extraction of CIEDs containing atrial lead (89.3% vs. 76.0%, *p* = 0.0235) or VDD lead (12.5% vs. 1.1%, *p* < 0.0001) was associated with a higher risk of LB. Figure 4b. Conversely, the presence of a high voltage lead was associated with protection against LB (25.0% vs. 40.2%, *p* = 0.0249). A higher number of leads in the extracted system was associated with a higher LB probability (2.4 ± 0.8 vs. 2.0 ± 0.7, *p* = 0.0002), as well as a higher number of abandoned leads (0.4 ± 0.6 vs. 0.1 ± 0.3, *p* < 0.0001). Figure 4c. Complete data are listed in Table S3.

### 3.5. Conduction of the TLE Procedure

A higher number of leads intended for extraction was correlated with a more frequent LB (2.4 ± 0.8 vs. 1.9 ± 0.7, *p* = 0.0001). Among the various TLE techniques we used, there was no LB during simple traction (0.0% vs. 26.0%, *p* < 0.0001) but necessity of the use of particular instruments specific for TLE like locking stylet (98.2% vs. 71.2%, *p* <0.0001), single traction with any of mechanical sheath systems (96.4% vs. 73.7%, *p* = 0.0001), mechanical non-powered telescopic sheaths (67.9% vs. 53.4%, *p* = 0.0373), powered rotational mechanical sheaths (44.6% vs. 29.3%, *p* = 0.0165), side loop (35.7% vs. 5.3%, *p* < 0.0001), or simple loop (33.9% vs. 2.2%, *p* < 0.0001) was associated with increased frequency of LB.

As the procedure duration increased, the likelihood of LB also increased (145.5 ± 80.9 vs. 84.8 ± 58.4 min, *p* < 0.0001). Complete data are listed in Table S4.

### 3.6. Complications Coexisting with a Lead Break

The event of LB is associated with a higher frequency of acute renal failure (7.1% vs. 1.5%; *p* = 0.0041) and pericardial effusion requiring intervention (5.4% vs. 1.4%; *p* = 0.0283). LB was not associated with periprocedural death. Complete data are listed in Table S5.

### 3.7. Mortality and Procedure Effects

Procedures ladened by LB were more likely to result in the retention of leads fragments (78.6% vs. 1.2%, *p* < 0.0001) with a length of both less than 4 cm (53.6% vs. 0.0%, *p* < 0.0001) and greater than 4 cm (25.0% vs. 1.2%, *p* < 0.0001), which, together with co-occurring complications, resulted into lower as procedural (57.16% vs. 94.4%, *p* < 0.0001) as clinical success (85.7% vs. 96.4%, *p* = 0.0002). LB did not significantly affect hospitalization duration or 30-day mortality. Complete data are listed in Table S6.

### 3.8. Predictive Model for Lead Break During TLE Procedure

Multivariate logistic regression analysis revealed independent predictors of LB during TLE. A higher patient age at TLE (*p* = 0.0432), as well as previous cardiac surgery (*p* = 0.0086) and later year of CIED implantation (*p* < 0.0001), were associated with a lower incidence of LB. VDD lead extraction (*p* = 0.0069) and an abandoned lead extraction procedure (*p* = 0.0261) were associated with a higher risk of lead break. The presence of an atrial lead (*p* = 0.0107) proved to be a protective factor. A trend of dependence was also demonstrated for the protective function of the locking stylet (*p* = 0.0795). The duration of TLE increases the risk of LB (*p* = 0.0001). Details of the prediction model obtained using multivariate logistic regression analysis are presented in Table 2.

## 4. Discussion

TLE is a widely accepted lead management method with a broad range of indications, including both infectious and non-infectious conditions. Due to the technique of the procedure, which involves the use of aggressive tools within the large vessels inside the chest and heart during the removal of leads that may be adhered to their walls, these procedures require utmost care and appropriate consideration [15]. Many authors have described the high effectiveness and safety of these procedures, supporting this with statistics on the incidence of typical complications in their centers. These are divided into major and minor, depending on their severity and reversibility. Major ones occur with a frequency of approximately 0.19–1.8%, while minor ones occur with a frequency of approximately 0.06–6.2% [7,16]. However, only a few publications describe the risk factors for complications of TLE procedures. LB is not a common or typical complication of the TLE procedure, but rather an event that increases the difficulty and complexity of the procedure and influences the likelihood of complications. We have identified two publications that analyzed the risk factors associated with this event. In our analysis, LB occurred during 7.98% of TLE procedures performed. A similar percentage was reported by Kutarski et al. (6.04%) [11], while in Morita et al.’s analysis, it was higher (20.1%). However, their definition of LB appears to include stretched leads too [10]. The tensile strength of the leads varies depending on the model and extraction technique used [17]. Knowing this helps to apply techniques that increase tensile strength [18].

**Figure 5:**
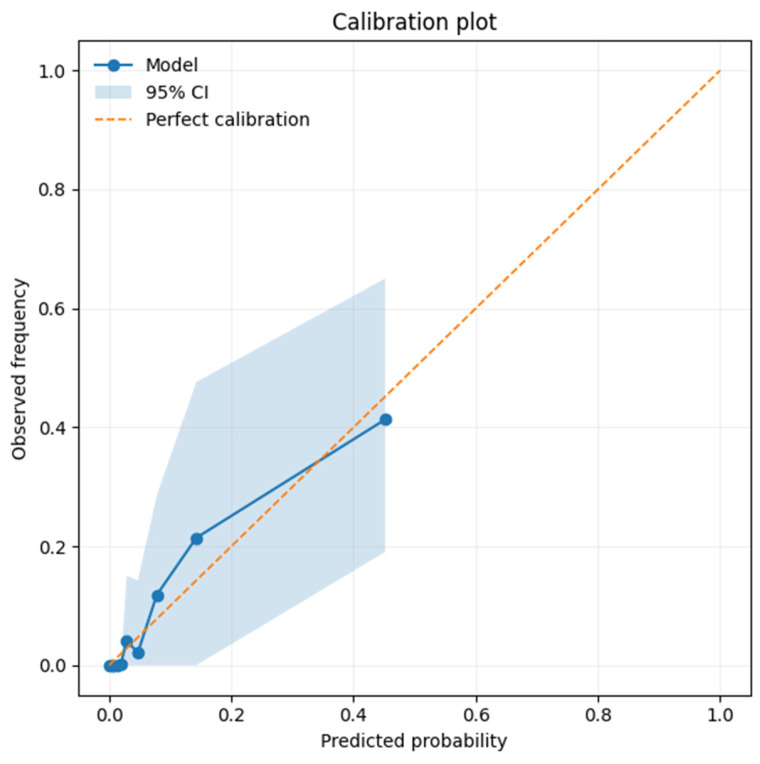
Calibration plot with bootstrap validation for the prediction model for lead breakage during TLE.

Multivariate logistic regression of our data yielded a well-fitting predictive model for LB occurrence, with an AUC of 0.899 (*p* < 0.0001) as confirmed by a Calibration Plot with Bootstrap validation (Figure 5). Predicted risk ranged from 0.0005 to 0.8359.

An intuitive parameter with a significant impact on LB incidence was the patient’s age at the time of procedure. However, in our data, younger patients, regardless of sex, experienced LB more frequently. We attribute this to a stronger inflammatory response to the foreign body, i.e., lead. A strong inflammatory response causes vessel occlusion, fibroblast activation, and increased ingrowth of lead into the vascular wall. Available publications are inconsistent regarding the validity of this mechanism [19]. In the analyses by Kutarski et al. and also Morito et al., age was not associated with the incidence of LB [10,11]. In Demian et al.’s analysis of the ExTRACT registry, no significant association was found between age and TLE complications. However, complete procedural success was the lowest in the youngest group, even if LB was not examined. The youngest group was categorized as <65 years old [20]. However, Cecchin et al. reported a lower success rate for simple traction TLE in pediatric patients [21].

The direct relationship between LB frequency and procedural time has a two-way explanation. A prolonged procedure may indicate that lead removal is challenging and may ultimately result in lead breakage. It is related to the higher complexity of TLE [11]. On the other hand, lead rupture necessitates a change in the procedure plan and the use of additional instrumentation, both of which are time-consuming and require extra effort.

Equipment manufacturers continually develop and improve their products while phasing out older ones. These include recalled leads, unipolar leads, passive leads, leads with a coradial structure or leads with non-ePTFE covers. We do not have complete data on the characteristics of leads subjected to TLE at our center in previous years. However, commercially available leads were implanted each year, and the analyzed parameter should be understood in this context. This finding is consistent with observations of other authors, who have reported that TLE of older lead types is associated with a higher rate of LB [10]. The date of first implantation, in fact, indicates the longest dwell time, a finding supported by the univariate analysis. Despite the limited number of studies describing LB, the association between lower complete procedural success of TLE and higher dwell time is well known [22,23,24].

VDD-type leads have a more complex design, featuring four channels—two for ventricular sense and pacing, and two for atrial sense. The atrial channel is completed with two floating rings in the atrial conduction of the lead body. Actually, these leads are not implanted in pulse generator devices, but only in cardioverter–defibrillators. Our data showed that this type of lead was more likely to break and increase the complexity of the procedure than other types. The same observation was made by Kutarski et al., who noted that it did not increase the major complication rate and could be correlated with specific patient characteristics associated with VDD leads [25]. Similar results were reported by Harunari et al., who found that VDD leads extraction resulted in a significantly reduced success rate [26].

Multivariate analysis demonstrated a lower LB rate during extraction procedures of CIED systems containing atrial leads. Kloppe et al.’s results confirm this observation, with the highest atrial lead extraction rate achieved with minimally invasive methods and the complete procedural success with TLE systems containing an atrial lead [27]. This is expected given the procedure’s design. Our experience dictates starting the extraction procedure with the strongest lead, and, if there is no progression or the lead body is stretched, continuing with the next lead. This often allows LB to be avoided and the first lead to be removed smoothly at a later stage.

The presence of abandoned leads in the system being extracted is associated with numerous complications, increasing the complexity of TLE, requiring the use of more advanced tools or multi-venous approaches, prolonging the procedure time, and increasing the risk of conversion to cardiothoracic surgery. This leads to a lack of procedural and clinical success with retention of a large portion of the leads [28,29,30]. The meta-analysis by Chauhan et al. [31] revealed that TLE of abandoned lead is associated with higher in-hospital mortality, major complications, and procedural failure rates compared to functional leads. Abandoned, non-functioning leads are typically older and have been previously damaged [6,28]. These leads are often found in veins that also contain a higher-than-usual number of leads, especially after undergoing multiple interventions. In such cases, the risk of LB is significantly increased.

The protective effect of cardiac surgery performed in the past for any reason is not intuitive. A more in-depth analysis of this group of patients revealed a preponderance of systems with high-voltage leads, which showed higher break resistance in univariate analysis. Pacemaker systems, which are more susceptible to tearing, were less widespread. Patients who had undergone surgery in the past also had a higher degree of heart failure, according to the NYHA scale, lower LVEF, and higher coronary artery disease burden. In the univariate analysis, such patients were less likely to experience LB.

Sometimes, simply tracking the lead is not enough for successful extraction. In such cases, it is standard practice to use a locking stylet. This device helps stiffen, strengthen, and lengthen the lead, preparing it for use with mechanical sheaths. Once the stylet is in place, the lead can endure greater forces and stresses, thereby reducing the likelihood of lead breakage [32,33].

The results of the analysis should be considered both when considering the indications for the procedure, as well as during the development of the procedure plan and the procedure itself. Besides the absolute indication for TLE, which is CIED-related infection, it should be remembered that in other indications, discarding unused or damaged leads and implanting a new one may be considered, depending on the expected risk. Particular attention should be paid to non-modifiable predictors influencing procedural and clinical success, such as age, year of implantation, artery, VVD, or abandoned leads. Furthermore, the analyzed parameters may have different significance depending on the endpoint being analyzed. For example, younger age increases the risk of LB, while older age increases the risk of death [34].

We propose the new acronym “**A LACY ART**” to summarize all proven predictors: **A**—age, **L**—locking stylet, **A**—atrial lead, **C**—cardiac surgery, **Y**—year of implantation (protective factors); **A**—abandoned lead, **R**—right ventricular VDD lead, **T**—time of procedure (risk factors).

## 5. Conclusions

Lead breakage is an underestimated procedural difficulty during transvenous lead extraction. In this study, several clinical and procedural variables were independently associated with lead breakage. Abandoned leads, VDD leads, and prolonged procedure time were associated with increased risk, whereas older age, use of a locking stylet, atrial lead extraction, prior cardiac surgery, and later year of implantation demonstrated an independent protective association.

### Limitations

Despite the large volume of TLE procedures performed, this is a single-center analysis. It should be noted that all procedures in our registry were performed using mechanical tools only, and the results cannot be projected to procedures performed using laser sheaths. The use of individual extraction techniques and tools was not randomized but was determined by clinical indications and procedural difficulties.

## Figures and Tables

**Table 1:** Demographics and medical history with univariate comparison between broken lead and non-broken lead during TLE patients.

Variable	The Entire Group, *n* = 702 Patients	The Broken Lead During the TLE Group, *n* = 56 Patients	The Non-Broken Lead During the TLE Group, *n* = 646 Patients	*p*-Value
Age (years)	66.2 ± 13.0	60.5 ± 15.8	66.7 ± 12.6	0.0079
Sex	F—245 (34.9%) M—457 (65.1%)	F—21 (37.5%) M—35 (62.5%)	F—224 (34.7%) M—422 (65.3%)	0.6707
Weight (kg)	81.0 ± 16.4	81.0 ± 16.1	81.0 ± 16.4	0.9344
Growth (cm)	168.5 ± 9.7	167.5 ± 15.6	168.6 ± 9.0	0.7532
Body Mass Index	28.4 ± 5.2	28.3 ± 6.0	28.4 ± 5.2	0.4043
Coronary artery disease, n (%)	359 (51.1%)	21 (37.5%)	338 (52.3%)	0.0334
Previous myocardial infarction, n (%)	240 (34.2%)	11 (19.6%)	229 (35.4%)	0.0168
Previous PCI, n (%)	229 (32.6%)	10 (17.9%)	219 (33.9%)	0.0141
Previous CABG, n (%)	100 (14.2%)	1 (1.8%)	99 (15.3%)	0.0055
Sum of CAD/MI/PCI/CABG (1 pt/any, max 4)	1.3 ± 1.5	0.8 ± 1.2	1.4 ± 1.5	0.0046
Previous cardiac surgery, n (%)	155 (22.1%)	3 (5.4%)	152 (23.5%)	0.0017
Pulmonary disease, n (%)	68 (9.7%)	2 (3.6%)	66 (10.2%)	0.1070
Peripheral artery disease, n (%)	185 (26.3%)	8 (14.3%)	177 (27.4%)	0.0327
Atrial fibrillation, n (%)	306 (43.6%)	17 (30.4%)	289 (44.7%)	0.0375
Arterial hypertension, n (%)	474 (67.5%)	30 (53.6%)	444 (68.7%)	0.0202
Chronic kidney disease, n (%)	171 (24.4%)	11 (19.6%)	160 (24.8%)	0.3917
Dependence on dialysis, n (%)	14 (2.0%)	1 (1.8%)	13 (2.0%)	0.9074
Diabetes mellitus, n (%)	214 (30.5%)	12 (21.4%)	202 (31.3%)	0.1252
Diabetes mellitus requiring insulin, n (%)	47 (6.7%)	1 (1.8%)	46 (7.1%)	0.1257
Previous stroke, n (%)	45 (6.4%)	4 (7.1%)	41 (6.3%)	0.8133
History of cancer disease, n (%)	50 (7.1%)	3 (5.4%)	47 (7.3%)	0.5926
Anticoagulant drugs used before TLE, n (%)	321 (45.7%)	19 (33.9%)	302 (46.7%)	0.0649
Antiplatelet drugs used before TLE, n (%)	206 (29.3%)	13 (23.2%)	193 (29.9%)	0.2939
NYHA class (0–IV)	1.1 ± 1.1	0.7 ± 1.1	1.1 ± 1.1	0.0016
Left ventricle ejection fraction (%)	45.3 ± 15.5	51.5 ± 13.7	44.8 ± 15.5	0.0018
TLE due to infection-related indications, n (%)	209 (29.8%)	14 (25.0%)	195 (30.2%)	0.4159
Pacing dependency, n (%)	134 (19.1%)	13 (23.2%)	121 (18.8%)	0.4164

CAD—coronary artery disease; MI—previous myocardial infarction; PCI—percutaneous coronary intervention; CABG—coronary artery bypass grafting; TLE—transvenous lead extraction; NYHA class—New York Heart Association class; Green: *p*-value < 0.05; Yellow: *p*-value 0.1–0.05

**Table 2:** Model for predicting lead breakages during the TLE.

Independent Predictors	Coefficient	Std. Error	Wald	*p*-Value	Odds Ratio	95% CI
Age (years)	−0.022580	0.011166	4.0889	0.0432	0.9777	0.9565 to 0.9993
Procedure time (min)	0.0081698	0.0021078	15.0232	0.0001	1.0082	1.0040 to 1.0124
Date of first implantation (year)	−0.10557	0.020632	26.1846	<0.0001	0.8998	0.8641 to 0.9369
Right ventricular as a VDD lead	2.00441	0.74131	7.3109	0.0069	7.4217	1.7357 to 31.7337
Atrial lead	−1.55496	0.60924	6.5143	0.0107	0.2112	0.0640 to 0.6971
Non-functional lead (abandoned)	0.74238	0.33378	4.9469	0.0261	2.1009	1.0922 to 4.0414
Previous cardiac surgery	−1.69915	0.64694	6.8981	0.0086	0.1828	0.0514 to 0.6498
Locking stylet use	−1.81485	1.03483	3.0757	0.0795	0.1629	0.0214 to 1.2379
Constant	210.34962	41.40256	25.8124	<0.0001		
Overall Model Fit *p* < 0.0001						
Hosmer–Lemeshow test		Chi-squared = 10.8185, *p* = 0.2122				
ROC curve analysis						
Area under the ROC curve (AUC)		0.899				
Significance level *p* (area = 0.5)		<0.0001				
Standard Error		0.0212				
95% Confidence Interval		0.874 to 0.920				
Sensitivity		83.93%				
Specificity		86.38%				
95% Bootstrap Confidence Interval		0.844 to 0.931				
Youden index J		0.7031				
Area under the ROC curve (AUC) ^a^		0.910				
95% Confidence Interval ^a^		0.847 to 0.958				
Brier score ^a^		0.055				
Brier score 95% Confidence Interval ^a^		0.034 to 0.079				
Predicted risk ^a^		0.0005 to 0.8359				

^a^ Performed using Python 3.14.2 with Pandas 2.3.3.

## Data Availability

The data that support the findings of this study are available from the corresponding author upon reasonable request. The study was conducted and reported in accordance with the TREND statement (Table S7) [35].

## Supplementary Materials

The following supporting information can be downloaded at: https://www.mdpi.com/article/10.3390/jcm15031216/s1, Table S1: Equipment used during transvenous lead extraction. Table S2: Laboratory parameters with univariate comparison between broken lead and non-broken lead during TLE patients. Table S3: Characteristics of systems being extracted with univariate comparison between broken lead and non-broken lead during TLE patients. Table S4: Techniques and conduct of the TLE procedure with univariate comparison between broken and non-broken leads during TLE patients. Table S5: Coexisting complications in the operative or postoperative period, with a univariate comparison between broken lead and non-broken lead in the TLE patients. Table S6: Mortality and operative or postoperative effects with univariate comparison between broken lead and non-broken lead during TLE patients. Table S7: TREND Statement Checklist.

## Abbreviations

The following abbreviations are used in this manuscript:

CABG	Coronary artery bypass grafting
CIED	Cardiac implantable electronic device
LB	Lead breakage
NYHA	New York Heart Association
PCI	Percutaneous coronary interventions
TEE	Transesophageal echocardiogram
TLE	Transvenous lead extraction
